# Exquisite ligand stereoselectivity of a *Drosophila* juvenile hormone receptor contrasts with its broad agonist repertoire

**DOI:** 10.1074/jbc.RA118.005992

**Published:** 2018-11-19

**Authors:** Lenka Bittova, Pavel Jedlicka, Martin Dracinsky, Palani Kirubakaran, Jiri Vondrasek, Robert Hanus, Marek Jindra

**Affiliations:** From the ‡ Biology Center of the Czech Academy of Sciences, Institute of Entomology, Ceske Budejovice 370 05, Czech Republic and; § Institute of Organic Chemistry and Biochemistry of the Czech Academy of Sciences, Prague 166 10, Czech Republic

**Keywords:** juvenile hormone (JH), hormone receptor, insect, Drosophila, basic helix-loop-helix transcription factor (bHLH), ligand-binding protein, stereoselectivity, development, reproduction

## Abstract

The sesquiterpenoid juvenile hormone (JH) is vital to insect development and reproduction. Intracellular JH receptors have recently been established as basic helix-loop-helix transcription factor (bHLH)/PAS proteins in *Drosophila melanogaster* known as germ cell–expressed (Gce) and its duplicate paralog, methoprene-tolerant (Met). Upon binding JH, Gce/Met activates its target genes. Insects possess multiple native JH homologs whose molecular activities remain unexplored, and diverse synthetic compounds including insecticides exert JH-like effects. How the JH receptor recognizes its ligands is unknown. To determine which structural features define an active JH receptor agonist, we tested several native JHs and their nonnative geometric and optical isomers for the ability to bind the *Drosophila* JH receptor Gce, to induce Gce-dependent transcription, and to affect the development of the fly. Our results revealed high ligand stereoselectivity of the receptor. The geometry of the JH skeleton, dictated by two stereogenic double bonds, was the most critical feature followed by the presence of an epoxide moiety at a terminal position. The optical isomerism at carbon C11 proved less important even though Gce preferentially bound a natural JH enantiomer. The results of receptor-ligand–binding and cell-based gene activation assays tightly correlated with the ability of different geometric JH isomers to induce gene expression and morphogenetic effects in the developing insects. Molecular modeling supported the requirement for the proper double-bond geometry of JH, which appears to be its major selective mechanism. The strict stereoselectivity of Gce toward the natural hormone contrasts with the high potency of synthetic Gce agonists of disparate chemistries.

## Introduction

Arthropods possess two major types of lipophilic hormones: steroids, mainly represented by ecdysone and its active form 20-hydroxyecdysone (20E),^4^ and the sesquiterpenoid juvenile hormones (JHs). In insects, 20E promotes metamorphosis from larvae to adults, whereas JH acts antagonistically to prevent the metamorphosis (1). In adult females of most insect species, JH has another discrete major role in promoting reproductive maturity and oogenesis (2). 20E activates the ecdysone receptor, a well-characterized member of the nuclear receptor family (3, 4). In contrast, the function of an intracellular receptor of JH has rather recently been ascribed to the methoprene-tolerant (Met) protein and its *Drosophila melanogaster* ancestral paralog, germ cell–expressed (Gce) (5–7). Met and Gce are members of the basic-helix-loop-helix (bHLH)/Per-Arnt-Sim (PAS) protein family (8). bHLH-PAS proteins form dimeric transcription factors, and some are activated by low-molecular-weight ligands (9). This applies to the vertebrate Aryl hydrocarbon receptor, which responds to endogenous ligands as well as environmental pollutants (10, 11), and to the insect Met/Gce. To date, Met/Gce remains the only known bHLH-PAS receptor for an authentic animal hormone (7). Importantly, Met/Gce is also activated by a large number of synthetic JH-mimicking compounds of variable chemistries, such as methoprene and pyriproxyfen (5, 6, 12–14), which are commonly used as insecticides disrupting insect development (15, 16).

Binding of JH or of its mimics to a hydrophobic pocket within the C-terminal PAS domain (PAS-B) of Met/Gce (5, 6, 17, 18) stimulates formation of a JH receptor complex with another bHLH-PAS protein, Taiman (Tai; also known as steroid receptor coactivator) (5, 12, 19). The complex binds DNA at specific JH response elements (JHREs) to activate transcription (12, 13, 18). The known genes directly controlled by the JH receptor include the repressor of insect metamorphosis, Krüppel homolog 1 (*Kr-h1*) (13, 20–24), and *early trypsin*, important for blood digestion in female mosquitoes (12, 18). To prepare the mosquito female for reproduction, JH receptor signaling directly or via downstream transcription factors orchestrates expression of large sets of genes (25, 26).

JH signaling clearly is vital to arthropods and important for our capacity to control insect pests and disease vectors by means of JH mimics. However, very little is known about how natural JHs or their synthetic agonists interact with the intracellular receptor and what decides which compound will or will not activate it.

**Figure 1.**
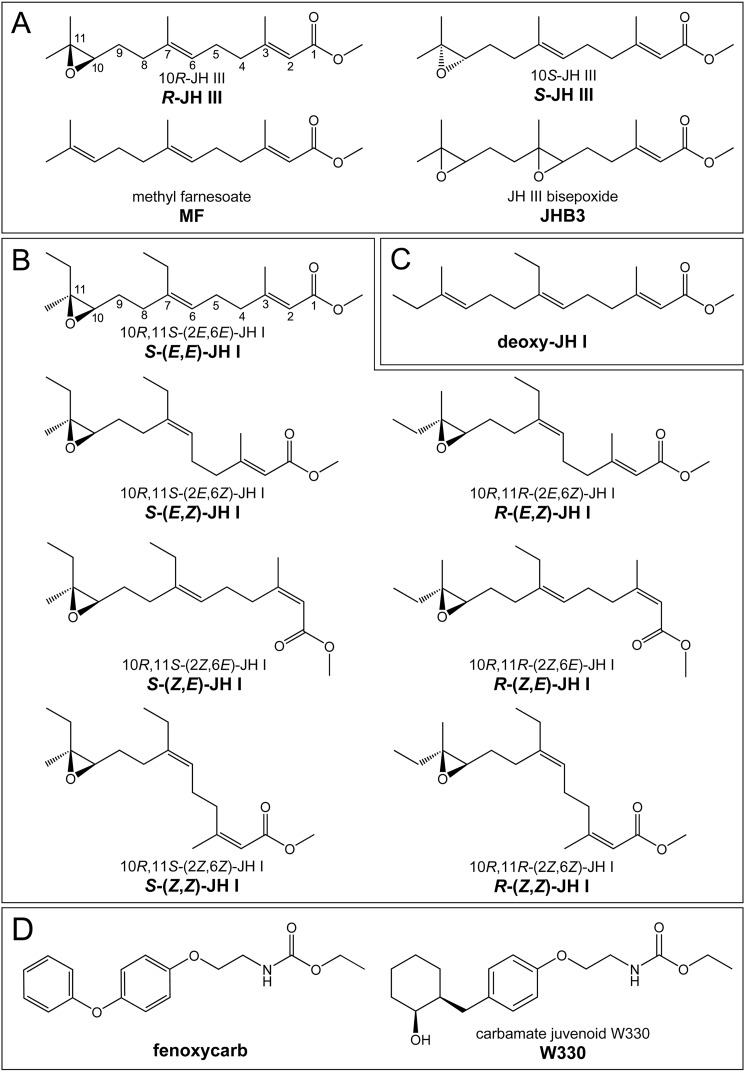
**Structures and names of the tested compounds.** *A*, three known native juvenile hormones in *D. melanogaster* and the unnatural 10*S*-JH III enantiomer. *B*, the native conformation of JH I (*top left*) and its geometric stereoisomers in both 10*R*,11*S* and 10*R*,11*R* configurations. *C*, methyl (2*E*,6*E*,10*E*)-7-ethyl-3,11-dimethyltrideca-2,6,10-trienoate, a nonepoxidated version of JH I, abbreviated as deoxy-JH I. *D*, carbamate-derived JH mimics. The abbreviated nomenclature (in *bold*) is preferentially used throughout this study.

Diverse groups of arthropods synthesize different types of JH (15). Crustaceans use methyl farnesoate (MF), a nonepoxidated biosynthetic precursor of insect JH (27). The main insect JH homologs differ by the numbers and positions of carbons and epoxide groups (see Fig. 1). JH III prevails in most insect taxa. JH I, which is only encountered in the Lepidoptera (*e.g.* moths) differs from JH III by the presence of ethyl instead of methyl groups at the C7 and C11 carbons of the sesquiterpenoid backbone (see Fig. 1). Double-epoxidated JHs have been found in certain true bugs (Heteroptera) (28) and some advanced Diptera (flies) (29, 30). Multiple JHs may even coexist as functional circulating hormones within one species as is the case of JH III, its bisepoxide variant JHB3, and MF (see Fig. 1) in *D. melanogaster* (29, 31, 32). However, functional differences between the chemical variants of the native JHs remain unclear.

JHs naturally synthesized by insect endocrine cells are 2*E*,6*E* geometric isomers (33). JH I has the absolute configuration 10*R*,11*S* at the C10 and C11 chiral centers (34) (see Fig. 1 and Fig. S1). The natural JH III with its single chiral center is the 10*R* enantiomer (35). Early studies have tested JH I stereoisomers for the capacity to inhibit insect metamorphosis (33, 36–40). Bioassays in beetle, moth, and heteropteran species generally concluded that the native spatial conformation of JH I was the most potent conformation and that the geometric isomerism had a great impact on the biological activity, whereas the chirality at C10 and C11 was less critical. These pharmacological data essentially corresponded to later studies on binding of the JH I and JH III stereoisomers to the hemolymph JH-binding proteins (hJHBPs) from several moth species (41–46). However, the impact of the stereoisomerism of JH on its gene regulatory function, mediated by receptors in the nucleus, has been waiting another four decades to be examined.

Having established the intracellular JH receptor Met/Gce (5, 6), we are finally in a position to address the long-standing questions regarding JH agonist selectivity. To this end, we have assessed diverse native JHs, their optical and geometric isomers, and two chemically unrelated JH mimics for their effects on the ligand–receptor interaction, transcriptional activation in a cell-based system and *in vivo*, and *Drosophila* development. Our findings reveal high ligand stereoselectivity of the JH receptor Gce, which contrasts with the disparate chemistry of highly potent synthetic JH mimics.

## Results

### Different native juvenile hormones bind and activate the Drosophila JH receptor Gce

**Figure 2.**
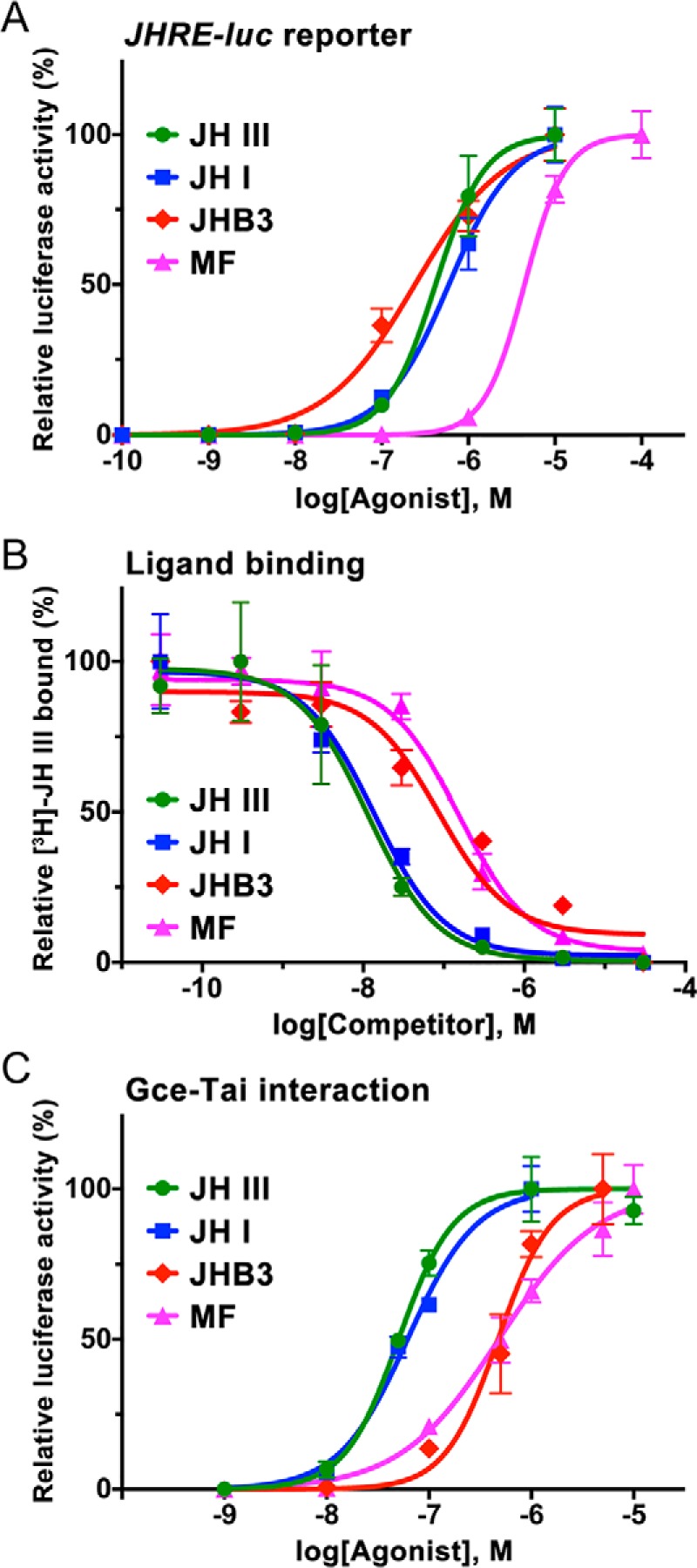
**Agonist activities of native insect JHs.** *A*, activation of the *JHRE-luc* reporter in S2 cells by three JHs known in *D. melanogaster* and by the lepidopteran JH I. The data (mean from three technical replicates) show a representative example of three or four independent experiments; *error bars* represent S.D. Average EC_50_ values were 0.21 ± 0.14 μm for *R*,*S*-JH III, 0.37 ± 0.22 μm for JH I, 0.11 ± 0.01 μm for JHB3, and 4.43 ± 0.95 μm for MF. MF was significantly less effective (*p* < 0.01, *t* test) than any of the three epoxidated hormones; differences between *R*,*S*-JH III and either JH I (*p* > 0.29; *t* test) or JHB3 (*p* > 0.37; *t* test) were not statistically significant. *B*, binding of the hormones to the Gce protein in competition assays with *R*,*S*-[^3^H]JH III. Data (mean with *error bars* representing S.D.) indicate average *K_i_* values of 11.0 ± 2.2 nm (*n* = 3) for *R*,*S*-JH III, 13.8 ± 5.1 nm (*n* = 5) for JH I, and 83.3 ± 40.8 nm (*n* = 3) for JHB3; a *K_i_* measured for MF (89.8 ± 26.7 nm) corresponds to a previously determined value (87.9 ± 22.2 nm) (6). Affinities were significantly different (*p* < 0.01, *t* test) between the two groups of compounds comprising *R*,*S*-JH III and JH I *versus* JHB3 and MF. *C*, two-hybrid assay in HEK293T cells reflecting ligand binding to the Gce protein. The data (mean from three technical replicates) show a representative example of three or four independent experiments; *error bars* represent S.D. Average EC_50_ values were 58.4 ± 16.9 nm for *R*,*S*-JH III, 53.4 ± 13.9 nm for JH I, 0.31 ± 0.15 μm for JHB3, and 0.30 ± 0.06 μm for MF. Differences were significant between the group comprising *R*,*S*-JH III and JH I *versus* JHB3 (*p* < 0.05, *t* test) and MF (*p* < 0.01, *t* test).

JH III, its precursor MF, and JHB3 (Fig. 1) are all considered circulating hormones in *D. melanogaster* (29, 31, 32, 47). Using a *Drosophila* Schneider 2 (S2) cell line, we have previously shown that MF and JH III (the commercial racemic mixture, hereafter referred to as *R*,*S*-JH III) induce a JH-responsive luciferase (*JHRE-luc*) reporter in a manner dependent on binding to the endogenous JH receptor Gce (6). Here, we also tested a double-epoxidated JHB3 (Fig. 1 and supporting information). The effective concentration (EC_50_) values for inducing the *JHRE-luc* reporter were roughly in the 100–200 nm range for *R*,*S*-JH III and JHB3, whereas MF was about 20–40-fold less potent (Fig. 2*A*). RNAi-mediated knockdown of Gce revealed that, like MF and JH III (6), JHB3 also induced the reporter in a Gce-dependent manner in the *Drosophila* S2 cells (Fig. S2).

To assess direct binding of the hormones to the Gce protein, we measured the inhibition constants (*K_i_*) of the ligands in competition against *R*,*S*-[^3^H]JH III. Although unlabeled *R*,*S*-JH III and JHB3 were both similarly more effective than MF in activating *JHRE-luc* in the S2 cells (Fig. 2*A*), JHB3 bound Gce with a higher *K_i_* (83.3 ± 40.8 nm) than did *R*,*S*-JH III (11.0 ± 2.2 nm) (Fig. 2*B*). Rather, the binding affinity of JHB3 to Gce was close to the *K_i_* established for MF (87.9 ± 22.2 nm) (6) (Fig. 2*B*). To address the discrepancy, we tested the hormones using an independent two-hybrid assay in a human (HEK293T) cell line where ligand binding to Gce is estimated from JH-dependent interaction between Gce and its partner protein, Tai (48). This experiment confirmed that both MF and JHB3 were indeed about 5-fold less potent than *R*,*S*-JH III in inducing the Gce–Tai dimerization (Fig. 2*C*). Why JHB3 was relatively more active in the *Drosophila* S2 cells than in the two ligand-binding assays is unclear, but it might be due to differences in solubility or stability of JHB3 in the different experiments.

The above results show that MF, JH III, and JHB3 all bind and activate the *Drosophila* JH receptor Gce, albeit with different efficiencies. When compared with closely related compounds lacking JH activity, such as the JH precursor farnesol (6) or JH isomers with altered geometry (see below), MF, JH III, and JHB3 were 2–3 orders of magnitude more effective Gce binders and *JHRE-luc* activators.

To test whether Gce can recognize a juvenile hormone of insects other than *Drosophila*, we used the lepidopteran type JH I (Fig. 1). Synthetic 10*R*,11*S*-(2*E*,6*E*)-JH I, which represents the native JH I configuration (Fig. S1), efficiently competed against *R*,*S*-[^3^H]JH III for binding to the Gce protein, reaching a *K_i_* of 13.8 ± 5.1 nm, similar to that of *R*,*S*-JH III (Fig. 2*B*). In the independent two-hybrid assay, JH I and *R*,*S*-JH III were also equally effective in stimulating the interaction between Gce and Tai (Fig. 2*C*). As expected based on these data, JH I activated the *JHRE-luc* reporter in the S2 cells with an EC_50_ that was not significantly different from the activity of *R*,*S*-JH III or JHB3 in the same assay (Fig. 2*A*).

Taken together, the above data indicate that *Drosophila* Gce is a functional receptor not only for the three endogenous JHs of the fly but also for the distinct lepidopteran JH I. The higher degree of epoxidation in JHB3 or the extra two carbons at the side chains of the JH I skeleton (Fig. 1) appear to have limited impact on the capacity of these native insect compounds as Gce agonists.

### Gce preferentially binds the natural JH III enantiomer

**Figure 3.**
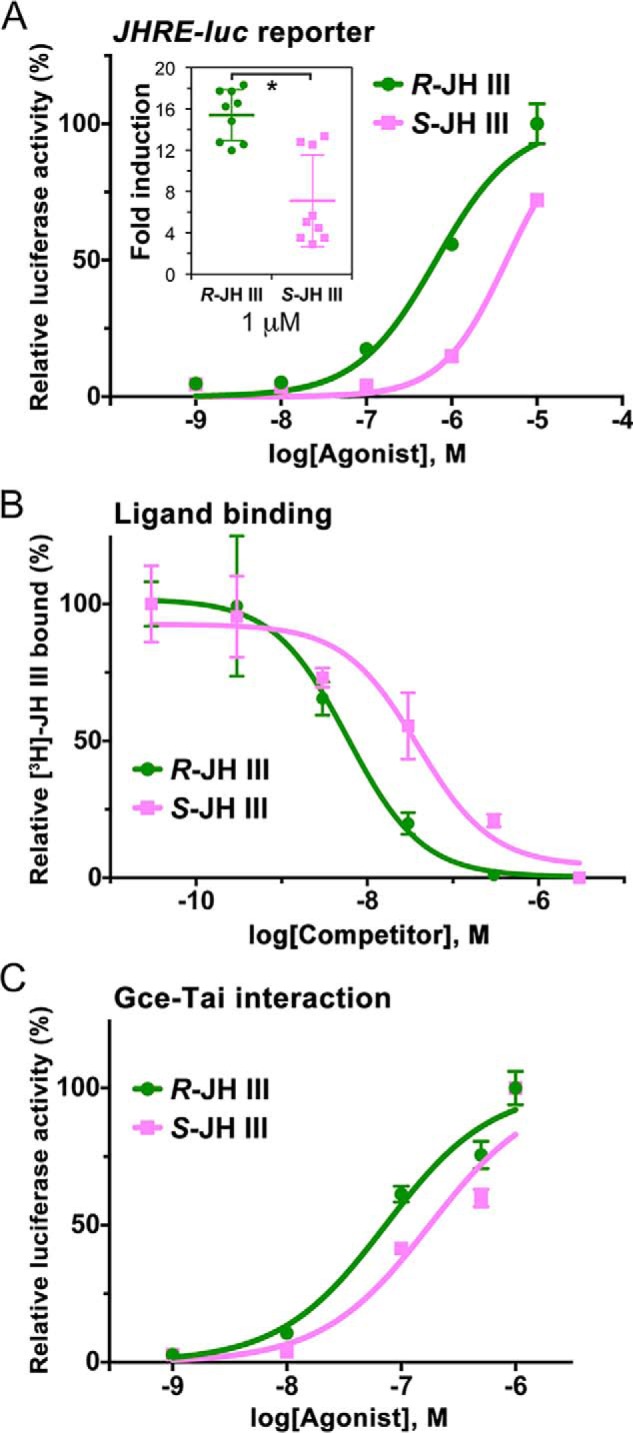
**Agonist activities of JH III enantiomers.** *A*, activation of the JH-inducible *JHRE-luc* reporter in the *Drosophila* S2 cell line. The data (mean from three technical replicates) show a representative example of three independent experiments. Although calculated EC_50_ values (0.93 ± 0.38 μm for *R*-JH III and 4.46 ± 2.95 μm for *S*-JH III) indicated about a 4-fold lower activity of the latter enantiomer, the difference was not statistically significant. Comparison of *JHRE-luc* expression at 1 μm concentration (*inset*) revealed a 2.2-fold higher activity of *R*-JH III (*, *p* < 0.05, *t* test). The normalized luciferase activities are plotted as -fold increase relative to treatment with solvent alone for which the value is arbitrarily set to 1. *B*, binding of *R*-JH III and *S*-JH III to the Gce protein *in vitro* as assessed from competition against racemic *R*,*S*-[^3^H]JH III. The data (mean from three measurements for each compound) revealed binding affinities (*K_i_* values) of 4.8 ± 1.3 and 38.3 ± 5.2 nm (*p* < 0.001, *t* test) for *R*-JH III and *S*-JH III, respectively. *C*, ligand binding–dependent interaction of Gce and Tai components of the JH receptor complex as assessed in a two-hybrid assay in HEK293T cells. The data (mean from three measurements) indicate EC_50_ values of 73.1 ± 5.2 and 169.8 ± 5.5 nm (*p* < 0.001, *t* test) for *R*-JH III and *S*-JH III, respectively. *Error bars* represent S.D. in all panels.

Although the insect corpora allata glands almost exclusively synthesize the 10*R*-epoxide enantiomer of JH III (49, 50), commercial preparations are racemic mixtures (50:50) of the 10*R* and 10*S* enantiomers (further referred to as *R*-JH III and *S*-JH III, respectively; Fig. 1). To test whether a JH receptor discriminates between the two optical isomers, we used enantiomerically pure *R*-JH III and *S*-JH III (51) in the *JHRE-luc* reporter assay in S2 cells. Although both enantiomers could elicit the specific transcriptional response, over a range of concentrations the unnatural *S* enantiomer was about 4-fold less effective than *R*-JH III in inducing the Gce-dependent activation of *JHRE-luc*, although its activity did not reach saturation (Fig. 3*A*). At 1 μm concentration, *R*-JH III induced *JHRE-luc* expression more than 2-fold higher relative to *S*-JH III (Fig. 3*A*, *inset*).

We next tested whether the stronger activity of *R*-JH III was reflected in direct ligand binding to the Gce protein. Indeed, Gce displayed an 8-fold higher binding affinity (*K_i_* = 4.8 ± 1.3 nm) to the natural *R* enantiomer relative to *S*-JH III (*K_i_* = 38.3 ± 5.2 nm) in the competition assay against *R*,*S*-[^3^H]JH III (Fig. 3*B*). The preference of Gce toward *R*-JH III, albeit less pronounced, was confirmed in the two-hybrid assay in HEK293T cells, which revealed that *R*-JH III (EC_50_ = 73.1 ± 5.2 nm) was ∼2.3-fold more effective than *S*-JH III (EC_50_ = 169.8 ± 5.5 nm) in stimulating the Gce–Tai interaction (Fig. 3*C*).

The three independent cell-based and ligand-binding assays yielded congruent data, supporting the expectation that the natural *R*-JH III enantiomer should be a better agonist than its optical antipode. However, the differences were relatively minor as even *S*-JH III attained appreciable activities in all assays (Fig. 3, *A–C*), suggesting that the exact configuration at the epoxide group is not a critical requirement for JH III to activate its intracellular receptor.

### The precise geometry of JH is critical for its agonist function

To address the importance of configuration of the double bonds in the JH backbone, we used a set of synthetic geometric isomers of 10*R*,11*S*-JH I, assuming the native 2*E*,6*E* and all three alternative *E*/*Z* configurations, hereafter referred to as *S*-(*E*,*Z*)-JH I, *S*-(*Z*,*E*)-JH I, and *S*-(*Z*,*Z*)-JH I, respectively (Fig. 1 and supporting information). In addition, we tested the altered double-bond geometry using isomers derived from the unnatural 10*R*,11*R* absolute configuration of JH I, designated *R*-(*E*,*Z*)-JH I, *R*-(*Z*,*E*)-JH I, and *R*-(*Z*,*Z*)-JH I (Fig. 1; see supporting information for NMR data). To assess the activities of the individual JH I geometric isomers as JH receptor agonists, we determined five parameters for each isomer, namely (i) affinity of binding to the Gce protein *in vitro*, (ii) ligand-induced interaction between Gce and Tai in the two-hybrid assay, (iii) activation of the *JHRE-luc* reporter in S2 cells, (iv) transcriptional induction of the JH-response *Kr-h1* gene *in vivo*, and (v) the capacity to affect *Drosophila* development.

**Figure 4.**
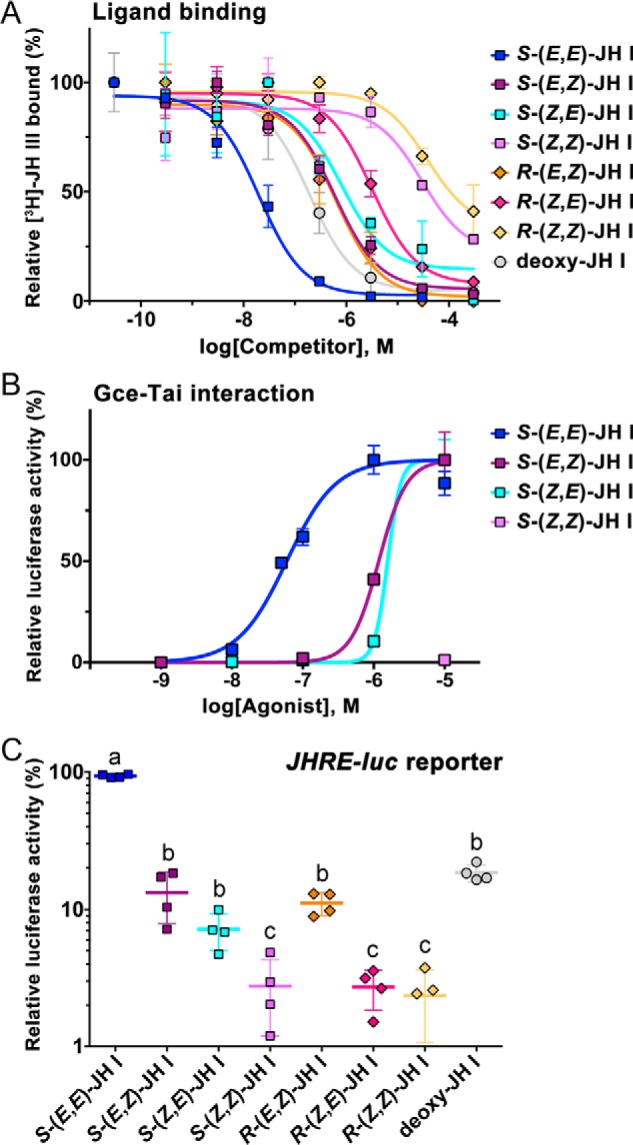
**Agonist activities of geometric isomers of JH I.** *A*, binding of the native *S*-(*E*,*E*)-JH I, its stereoisomers, and nonepoxidated deoxy-JH I to the Gce protein *in vitro* as assessed from competition against *R*,*S*-[^3^H]JH III. The data (mean from three measurements for each compound) revealed the binding affinities (*K_i_* values) listed in Table 1. *B*, ligand binding–dependent Gce–Tai dimerization in the two-hybrid assay in HEK293T cells. The data (mean from three technical replicates) show a representative example of three independent experiments that together indicated EC_50_ values of 53.4 ± 13.9 nm for *S*-(*E*,*E*), 1.24 ± 0.09 μm for *S*-(*E*,*Z*), and 1.60 ± 0.04 μm for *S*-(*Z*,*E*) JH I isomers; *S*-(*Z*,*Z*)-JH I was inactive. *C*, induction of *JHRE-luc* in the *Drosophila* S2 cell-based reporter assay by the indicated compounds at 1 μm concentration. The data are mean values from four independent experiments (each in three technical replicates). Different *letters* above the data indicate that activity of the individual compounds differed significantly (*p* < 0.05) as determined by one-way analysis of variance of log_10_-transformed data with Tukey's multiple post hoc comparison. *Error bars* represent S.D. in all panels.

As already shown above (Fig. 2*B*), the native *S*-(*E*,*E*)-JH I isomer bound avidly to Gce (*K_i_* = 13.8 nm, inferred from competition against *R*,*S*-[^3^H]JH III). Altering the configuration at either one of the double bonds in JH I reduced this affinity ∼32- and 45-fold, respectively, as determined in the same binding assay for the *S*-(*E*,*Z*)-JH I and *S*-(*Z*,*E*)-JH I isomers (Fig. 4*A* and Table 1). When the geometry turned opposite to the native geometry at both positions, *S*-(*Z*,*Z*)-JH I no longer efficiently competed against *R*,*S*-[^3^H]JH III in Gce binding; its apparent affinity dropped about 1640-fold (*K_i_* = 22.6 μm) relative to *S*-(*E*,*E*)-JH I (Fig. 4*A* and Table 1). This value was near a *K_i_* (8.1 ± 2.4 μm) previously reported for the biologically inactive JH precursor farnesol (6). In terms of binding affinity to Gce, the geometric isomers of 10*R*,11*S*-JH I therefore ranked *E*,*E* (native) ≫ *E*,*Z* > *Z*,*E* ≫ *Z*,*Z*.

**Table 1:** **Binding affinities of JH ligands to the Gce protein and Gibbs (ΔG) and ligand strain energies**

Compound	Affinity of ligand binding to Gce (*K_i_*)*^a^*	Δ*G* of ligand binding	Ligand strain energy
	*m*	*kcal mol*^−*1*^	*kcal mol*^−*1*^
*R*-JH III	1.10 × 10^−8^*^b^*	−108.70	8.44
*S*-(*E*,*E*)-JH I*^c^*	1.38 × 10^−8^	−137.80	11.16
*S*-(*E*,*Z*)-JH I	4.47 × 10^−7^	−130.42	9.72
*R*-(*E*,*Z*)-JH I	4.79 × 10^−7^	−122.24	17.88
*S*-(*Z*,*E*)-JH I	6.14 × 10^−7^	−119.60	21.41
*R*-(*Z*,*E*)-JH I	2.40 × 10^−6^	−109.10	25.18
*S*-(*Z*,*Z*)-JH I	2.26 × 10^−5^	−106.65	24.22
*R*-(*Z*,*Z*)-JH I	2.89 × 10^−5^	−100.16	24.52

*^a^* Determined from competition against *R*,*S*-[^3^H]JH III binding to the Gce protein.

*^b^ K_i_* determined for the racemic mixture of 10*R* and 10*S* enantiomers of (2*E*,6*E*)-JH III.

*^c^* Native hormone with the absolute configuration 10*R*,11*S*-(2*E*,6*E*)-JH I.

Similar data were obtained from the two-hybrid assay of JH-induced Gce–Tai interaction. The native *S*-(*E*,*E*)-JH I elicited this interaction with an EC_50_ of 53.4 ± 13.9 nm, whereas *S*-(*E*,*Z*) and *S*-(*Z*,*E*)-JH I ranked second and third by being ∼23- and 30-fold less effective; the *S*-(*Z*,*Z*)-JH I isomer was inactive (Fig. 4*B*).

Importantly, the same ranking was revealed in the *Drosophila* S2 cell–based assay where activation of the *JHRE-luc* reporter by JH I depends on Gce (Fig. S2). While native *S*-(*E*,*E*)-JH I was by far the best activator, comparable with *R*,*S*-JH III (Fig. 2*A*), the *S*-(*E*,*Z*)- and *S*-(*Z*,*E*)-JH I isomers induced strong *JHRE-luc* expression only at the highest, 10 μm dose, and *S*-(*Z*,*Z*)-JH I was ineffective even at this concentration (Fig. S3). Because *JHRE-luc* activation by the unnatural geometric isomers did not reach saturation, rather than EC_50_ we compared values at 1 μm concentration where *S*-(*E*,*Z*)- and *S*-(*Z*,*E*)-JH I achieved moderate induction (Fig. 4*C*).

To further corroborate these results, we tested *R*-(*E*,*Z*), *R*-(*Z*,*E*), and *R*-(*Z*,*Z*) geometric JH I isomers with the 10*R*,11*R* configuration (Fig. 1). Their binding affinities to the Gce protein, estimated from competition against *R*,*S*-[^3^H]JH III, again ranked the isomers in the established order *E*,*Z* > *Z*,*E* ≫ *Z*,*Z* (Fig. 4*A* and Table 1). Except for the greater difference of the third ranking *R*-(*Z*,*E*)- from the second ranking *R*-(*E*,*Z*)-JH I, the *K_i_* values of the 10*R*,11*R* isomers matched those determined for the corresponding 10*R*,11*S* homologs remarkably well (Table 1). Consistently, the *R*-(*E*,*Z*)-JH I isomer performed better than *R*-(*Z*,*E*)-JH I in the transcriptional activation of *JHRE-luc* in S2 cells (Fig. 4*C*). Although we did not have the 10*R*,11*R* optical isomer of (2*E*,6*E*)-JH I to directly assess the effect of the absolute configuration at the C11 chiral center, comparison of the Gce binding and transactivation data, particularly those obtained for *R*-(*E*,*Z*)-JH I and *S*-(*E*,*Z*)-JH I, suggests that, while the natural JH I enantiomer is a better agonist, the chirality is of secondary importance relative to the double-bond geometry.

### Activity of the geometric JH I isomers in vivo

**Figure 5.**
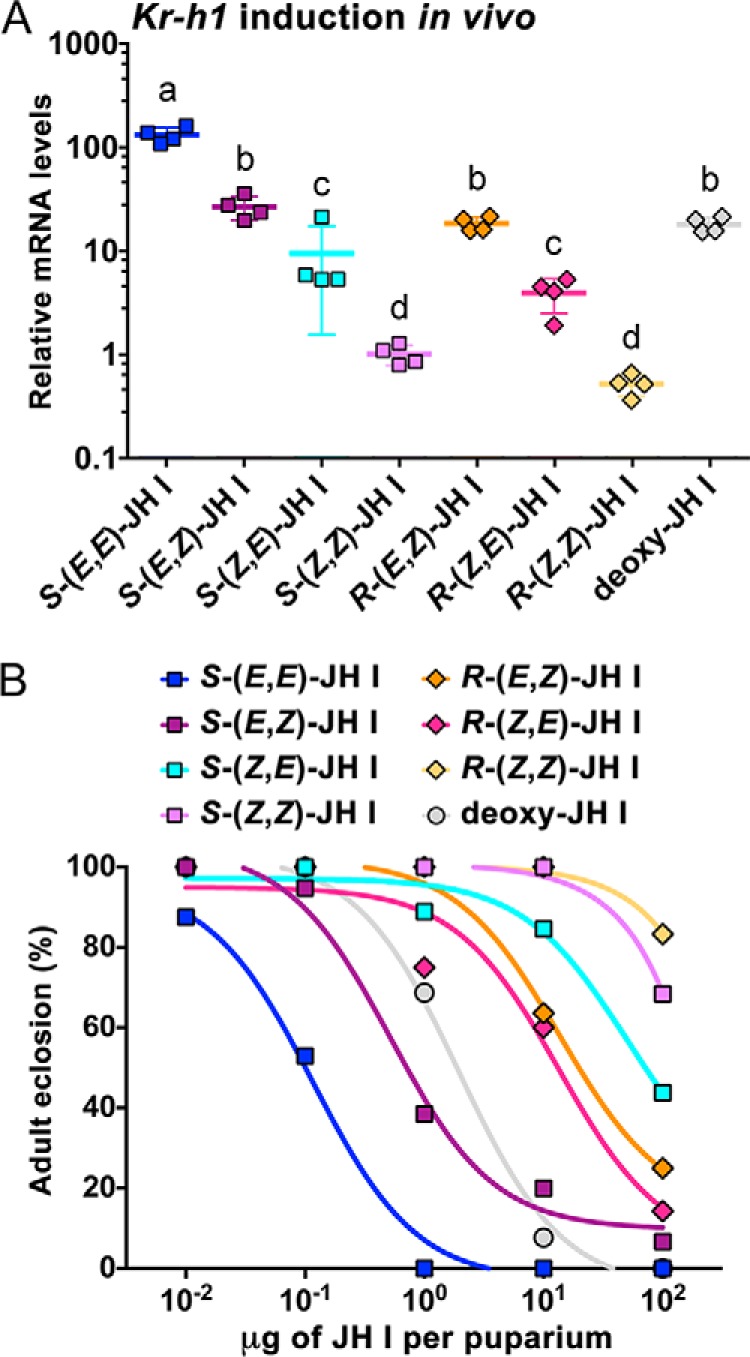
**Activity of the JH I geometric isomers *in vivo*.** *A*, capacity of the native *S*-(*E*,*E*)-JH I, its stereoisomers, and deoxy-JH I to induce ectopic expression of *Kr-h1* mRNA in *Drosophila* pupae. Animals were treated at the white puparium stage and collected 24 h later. Shown are normalized mean qRT-PCR data from four biological replicates, each comprising three individual pupae for each compound. The mRNA levels are plotted on a logarithmic scale as -fold increase relative to treatment with solvent (acetone) alone for which the value was arbitrarily set to 1. *Error bars* represent S.D. Different *letters* above the data indicate that activity of the individual compounds differed significantly (*p* < 0.05) as determined by one-way analysis of variance of log_10_-transformed data with Tukey's multiple post hoc comparison. *B*, effect of the JH I stereoisomers and deoxy-JH I on the ability of flies to complete development. Animals were again treated with the compounds as white puparia (*n* = 15–20 specimens per treatment).

To see whether the data from the *in vitro* binding and the cell-based assays agree with an authentic transcriptional response to JH, we chose the *Kr-h1* gene, which is directly induced by the ligand-activated JH receptor complex (6, 13, 18, 52). Because *Kr-h1* blocks *Drosophila* adult development, its transcription is normally suppressed in the pupal stage when endogenous JH is absent (20). Administration of the JH mimic pyriproxyfen to newly formed white puparia (12 h prior to pupation) causes a marked ectopic increase in *Kr-h1* mRNA throughout the pupal stage (20). We adopted this robust effect to measure the agonist potential of the JH I geometric isomers *in vivo*. The highest induction of the *Kr-h1* mRNA in pupae was achieved with the native *S*-(*E*,*E*)-JH I hormone followed by the *S*-(*E*,*Z*)-JH I and *R*-(*E*,*Z*)-JH I isomers that were 5- and 7.2-fold less effective, respectively (Fig. 5*A*). Alteration of the C2 double bond was more detrimental, leading to 14- and 34-fold reduction of activity, respectively, of the *S*-(*Z*,*E*)-JH I and *R*-(*Z*,*E*)-JH I isomers. Both *S*-(*Z*,*Z*)-JH I and *R*-(*Z*,*Z*)-JH I were virtually inactive, retaining less than 1% of the activity of the natural JH I (Fig. 5*A*). These *in vivo* data thus perfectly agreed with the capacity of the geometric isomers to bind the Gce protein and to activate the *JHRE-luc* reporter in *Drosophila* S2 cells (Fig. 4 and Table 1).

Application of JH or its mimics to white puparia of *Drosophila* disrupts adult fly development (20, 53, 54). Affected animals die as pupa-adult intermediates or fail to eclose as adults from the pupal case. We used this bioassay to score the effects of altered JH geometry on *Drosophila* development. Over the range of doses from 0.1 to 100 μg applied per puparium, the native *S*-(*E*,*E*)-JH I was about 5-fold more potent in preventing normal adult development than the second ranking *S*-(*E*,*Z*)-JH I (Fig. 5*B*). This result exactly matched the capacity of these two isomers to induce *Kr-h1* transcription in the treated pupae (Fig. 5*A*). By contrast, activity of the *R*-(*E*,*Z*)-, *R*-(*Z*,*E*)-, and *S*-(*Z*,*E*)-JH I isomers was more than 100-fold weaker compared with *S*-(*E*,*E*)-JH I. The two remaining *S*-(*Z*,*Z*)- and *R*-(*Z*,*Z*)-JH I compounds were essentially ineffective, causing failure to eclose in 32 and 17% adults, respectively, only at the highest tested dose (Fig. 5*B*). These results indicated that altering the double-bond geometry at either the C2 or both the C2 and C6 positions rendered JH I inactive in the white puparium bioassay.

### Molecular modeling of the activity of JH I isomers

**Figure 6.**
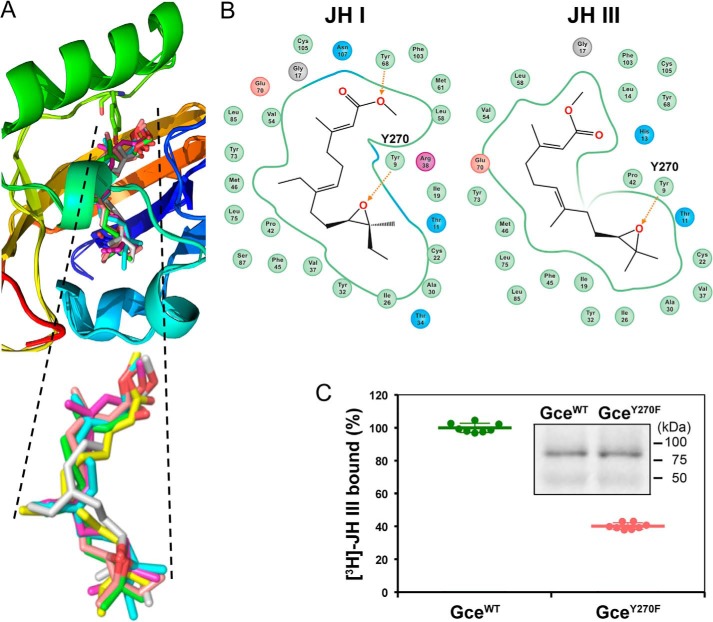
**Modeling of JH interaction with the Gce protein.** *A*, docking of the native conformations *R*-JH-III (*green*) and *S*-(*E*,*E*)-JH I (*cyan*) and of the JH I isomers *S*-(*E*,*Z*) (*yellow*), *S*-(*Z*,*E*) (*magenta*), *R*-(*E*,*Z*) (*gray*), and *R*-(*Z*,*E*) (*pink*) in the model of the PAS-B–binding pocket of *Drosophila* Gce. *B*, two-dimensional interaction diagrams of the native JH I (*left*) and JH III (*right*) with the PAS-B domain of *Drosophila* Gce. *Numbers* are amino acid positions within the modeled region; corresponding positions within the *Drosophila* Gce protein (NCBI Reference Sequence NP_511160.1) are the displayed numbers plus 261 (*i.e.* Tyr-9 is Tyr-270). *C*, loss of the hydroxyl group in the mutated Gce^Y270F^ protein reduced the amount of bound [^3^H]JH III to 40% of the WT (Gce^WT^) protein (*n* = 8; *p* < 4.83 × 10^−17^, *t* test). Data are mean values; *error bars* represent S.D. *Inset*, immunoblot of the *in vitro* expressed, Myc-tagged Gce proteins used in the binding assay.

To address the selectivity and binding mechanism of the JH receptor, we modeled the 3D structure of the Gce PAS-B domain and compared it with that of the PAS-B domain previously modeled for the Met ortholog from the beetle *Tribolium castaneum* (5). The structure alignment showed a superimposition of 0.58 Å of backbone atoms (Fig. S4). The 3D model revealed an α/β-fold with a unique α-helix between amino acids Asp-311 and Cys-325 in the *Drosophila* Gce protein (Asp-50 to Cys-64 of the modeled region), matching a homologous helix in *Tribolium* Met. The results indicate that JH receptors from both species share a similar structural fold (Fig. S4). The model of the Gce hormone–binding pocket was extended to dock the native JH III and JH I and the geometric isomers of JH I in both the normal 10*R*,11*S* and the unnatural 10*R*,11*R* absolute configurations. The docking identified bound states for all ligands with highly similar conformations within the binding pocket (Fig. 6*A*). The Δ*G* of binding for each ligand was determined using the MMGBSA (molecular mechanics, the generalized Born model and solvent accessibility) approach (see “Experimental procedures”), ranking the JH I ligands in the expected order with the most negative Δ*G* for the native hormone and increasing to the least negative values for the inactive *Z*,*Z* isomers (Table 1). The relatively positive Δ*G* calculated for JH III partly reflects a single hydrogen bond in the docking model of JH III rather than two hydrogen bonds predicted by docking of JH I (Fig. 6*B*).

The results of our modeling further suggest that binding scores not only capture the optimal ligand placement in the cavity but to a large extent also the internal conformational barriers between the bound and unbound states of the ligand. Indeed, with the exception of the moderately active *S*-(*E*,*Z*)-JH I, the calculated strain energies were the lowest for JH III and JH I in their natural conformations and the highest for the inactive *Z*,*Z* isomers (Table 1). Therefore, the docking studies identify a plausible and rational binding mode of most of the tested ligands and suggest that the energy required for the ligand conformational change is a major factor of the ligand selection toward the PAS-B–binding pocket of the JH receptor.

### Contribution of the epoxide moiety of JH to Gce binding

The position of the docked JH ligands in our Gce PAS-B model corresponds to the reported model of the *Tribolium* Met–JH III complex (5). Both models predict a hydrogen bond between the epoxide moiety of either JH I or JH III and the hydroxyl group of a conserved tyrosine residue (Tyr-270 in the *Drosophila* Gce protein; Tyr-9 within the modeled region) (Fig. 6*B*). To test the contribution of this hydrogen bond to JH binding by the Gce protein *in vitro*, we removed the hydroxyl group by mutating Tyr-270 to phenylalanine. The Gce^Y270F^ mutant retained 40% of the [^3^H]JH III binding capacity of the WT Gce protein (Fig. 6*C*). Consistent with this reduction, MF that lacks the epoxide and thus cannot form the hydrogen bond with Tyr-270 displayed a lower binding affinity to WT Gce relative to the affinity of JH III and was also less effective than JH III in inducing both the *JHRE-luc* reporter and the ligand dependent Gce–Tai interaction (Fig. 2, *A–C*).

To examine the impact of epoxidation loss on Gce binding to JH I, we used a compound that represents a nonepoxidated version of JH I, herewith referred to as “deoxy-JH I” (Fig. 1 and supporting information). With a 143.4 ± 45.0 nm *K_i_* assessed from competition against *R*,*S*-[^3^H]JH III, deoxy-JH I bound the Gce protein with an affinity about 10-fold lower than that of *S*-(*E*,*E*)-JH I (Fig. 4*A*). At 1 μm concentration, deoxy-JH I activated *JHRE-luc* in S2 cells to ∼17% of the level achieved with the native JH I (Fig. 4*C*). Consistent with these data, deoxy-JH I showed intermediate activities in inducing both the *Kr-h1* mRNA expression and developmental arrest in *Drosophila* pupae (Fig. 5, *A* and *B*).

### Activation of Gce by compounds chemically divergent from juvenile hormones

About 4000 compounds exerting JH-like effects on immature insects have been synthesized, some of which are used as insecticides (16, 40). It is of interest to know whether these chemically diverse compounds act through the same JH receptor as the native hormones. We have shown (6) that Gce binds methoprene, whose acyclic structure resembles that of native JHs. However, Gce also binds a pyridine derivative, pyriproxyfen, and both of these established insecticides are potent activators of the *JHRE-luc* reporter in *Drosophila* S2 cells (6).

**Figure 7.**
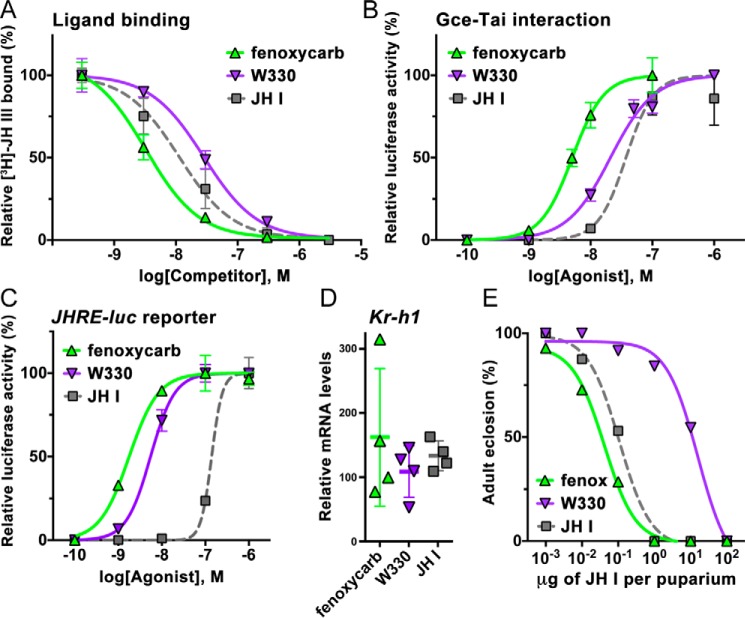
**Comparison of agonist activities between carbamate JH mimics and a native JH.** *A*, binding of the insecticide fenoxycarb and of the carbamate juvenoid W330 to the Gce protein determined in a competition assay with *R*,*S*-[^3^H]JH III. The data (mean with *error bars* representing S.D.) indicate a *K_i_* of 2.8 ± 1.1 nm (*n* = 3) for fenoxycarb, which differed significantly (*t* test) from the *K_i_* values of 13.8 ± 5.1 (*n* = 5) and 17.4 ± 5.7 nm (*n* = 4) determined for JH I (*p* < 0.05) and W330 (*p* < 0.01), respectively. The difference between W330 and JH I was not statistically significant. *B*, fenoxycarb and W330 were both significantly (*p* < 0.001, *t* test) more effective in stimulating Gce–Tai dimerization with EC_50_ values of 5.4 ± 0.8 (*n* = 4) and 17.7 ± 4.9 nm (*n* = 4), respectively. *Error bars* represent S.D. *C*, with EC_50_ values reaching 2.29 ± 0.65 (*n* = 4) and 5.42 ± 0.41 nm (*n* = 3), respectively, fenoxycarb and W330 were much stronger than JH I in activating the *JHRE-luc* reporter in *Drosophila* S2 cells. *Error bars* represent S.D. *D*, fenoxycarb, W330, and JH I all induced *Kr-h1* mRNA expression in *Drosophila* pupae to a similar degree (differences not statistically significant). Animals were treated at the white puparium stage and collected 24 h later. Shown are normalized mean qRT-PCR data from four biological replicates, each comprising three individual pupae; *error bars* represent S.D. The mRNA levels are plotted as -fold increase relative to treatment with solvent (acetone) alone for which the value was arbitrarily set to 1. *E*, capacity of the carbamates to prevent normal fly development as assessed in the white puparium bioassay (*n* = 15–20 specimens per treatment). *fenox*, fenoxycarb.

Here, we tested agonist capacities of another type of compounds, the bicyclic carbamate derivatives, represented by the insecticide fenoxycarb and a carbamate juvenoid, W330 (55) (Fig. 1). Fenoxycarb was the most effective ligand as it competed against *R*,*S*-[^3^H]JH III with a *K_i_* of 2.8 ± 1.1 nm for binding to the Gce protein *in vitro*; the affinity of W330 was ∼6-fold lower (*K_i_* = 17.4 ± 5.7 nm) and thus closer to the affinity of the native JH III or JH I (Fig. 7*A*). Both fenoxycarb and W330 were more effective than the native JHs in inducing the ligand-dependent interaction of Gce with Tai (Fig. 7*B*). The carbamates were also much stronger activators of the *JHRE-luc* reporter in S2 cells, reaching EC_50_ values of 2.3 ± 0.7 and 5.4 ± 0.4 nm for fenoxycarb and W330, respectively (Fig. 7*C*). RNAi knockdown experiments confirmed that, like the native JHs, fenoxycarb and W330 activated *JHRE-luc* expression through Gce rather than the alternate JH receptor Met (Fig. S2).

Consistent with the above results, both carbamates were highly active *in vivo*, and particularly fenoxycarb was at least as effective as JH I in inducing either *Kr-h1* mRNA expression or the morphogenetic defects in *Drosophila* pupae (Fig. 7, *D* and *E*). These data indicate that despite their chemistry, unrelated to that of JH, the synthetic carbamates are potent JH receptor agonists.

## Discussion

An intracellular receptor for JH had long remained elusive, which caused a major gap in our knowledge of how this versatile hormone directs insect life. Now that the receptor proteins are known, we have begun to bridge this gap by testing which structural features are critical for a JH to exert its agonist activity.

### The JH receptor Gce recognizes all Drosophila juvenile hormones and JH I

Our results show that three native JHs known in *D. melanogaster*, namely JH III, JHB3, and MF, bind Gce and activate its transcriptional response. Compared with JH III, the other two hormones, JHB3 and MF, have lower affinity to Gce, which might be compensated by higher titers of both JHB3 and MF in the hemolymph (56) or whole body (32) of *Drosophila* larvae than those of JH III. The lower activities of JHB3 and MF relative to JH III in our assays are consistent with previously reported effects on *Drosophila* development where the potency of the hormones ranked JH III > JHB3 > MF (16, 47). The same ranking applied to the ability to induce *Kr-h1* mRNA expression in a *Drosophila* Kc cell line (32).

The Gce receptor could not discriminate, in any of our assays, between the native *Drosophila* hormones and JH I, which is uniquely found in some lepidopterans. This is not surprising as the Met protein in a cell line from the beetle *T. castaneum*, a species that also uses JH III, responded equally well to either JH III or JH I by inducing *Kr-h1* expression (14). The presence of two ethyl groups in JH I (Fig. 1) increases the bulk of the JH I ligand to 516 Å^3^ relative to 460 Å^3^ for JH III (57). Nonetheless, the calculated volume (58) of the Gce PAS-B ligand–binding cavity is 556 Å^3^ and thus sufficient for the hydrophobic and probably flexible ligand-binding pocket to accommodate JH I. Interestingly, computational docking predicts some differences in the interaction of JH III and JH I with the Gce PAS-B domain (Fig. 6*C*).

### Enantioselectivity of Gce

Our competition ligand-binding and cell-based reporter assays show that the Gce receptor is moderately but consistently selective toward the natural 10*R*-JH III enantiomer, which is synthesized by the insect endocrine glands (49). The about 8-fold lower affinity of Gce toward 10*S*-JH III relative to the natural enantiomer correlates with a slightly more pronounced (14-fold) affinity of 10*R*-JH III for a hJHBP and its 12-fold greater biological activity in lepidopteran species (42). The natural (10*R*,11*S*) geometric isomer of JH I was only 2.5–3.5-fold more effective than its antipode in blocking metamorphosis in diverse insects (36, 38, 39). hJHBPs from several moths showed moderate preference for 10*R*,11*S*-JH I and 10*R*,11*S*-JH II *versus* the 10*R*,11*R* isomers (ratios of binding affinities ranging between 2- and 9-fold) (43–46). Similarly, 10*R*-JH III had 2.7-fold higher affinity than 10*S*-JH III for an mJHBP of another protein family found in mosquitoes where JH III is the native hormone (59). Together, these findings conform with our conclusion that the relatively high agonist activity of 10*S*-JH III indicates that the optical configuration is of minor importance for ligand selectivity of the JH receptor.

### Importance of the epoxide moiety

The epoxide ring connecting carbons 10 and 11 has always been considered an important functional feature of native insect JHs. Consistently, mutants of the *Bombyx mori* silkworms that cannot synthesize epoxidated JHs due to deficiency of a JH epoxidase pupate prematurely, suggesting that JH precursors such as MF cannot sustain normal development of *B. mori* (50). However, MF may not be released by the silkworm corpora allata, whereas in *D. melanogaster* MF is thought to be a circulating hormone (32, 56). MF is clearly less effective than JH I or JH III in inducing the transcriptional activity of either *Bombyx* or *Drosophila* JH receptors Met and Gce (6, 13) or their interaction with Tai as shown previously (48) and in this study. We obtained similar data with a nonepoxidated version of JH I, which was less potent than the native *S*-(*E*,*E*)-JH I hormone in all assays that reflect either Gce binding or transcriptional activation. Therefore, the absence of epoxide either from JH III (MF) or from JH I generally lowers the agonist activity by about 1 order of magnitude.

Structure resolution of hemolymph JH-binding proteins has provided a basis for the preference toward epoxidated and enantiospecific JHs (60). An NMR solution structure revealed a hydrogen bond forming between the hydroxyl group of Tyr-128 in the hJHBP of *B. mori* and the epoxide oxygen of JH III. Interestingly, a hydrogen bond was also found between the JH III epoxide and Tyr-129 in a crystal structure of an otherwise heterologous mJHBP of the mosquito *Aedes aegypti* (59). Although hJHBP, mJHBP, and the intracellular JH receptors Met/Gce are all unrelated proteins, our computational docking models also predicted a hydrogen bond between the epoxide oxygen of JH I or JH III and the hydroxyl group of Tyr-270 within the PAS-B pocket of Gce. This tyrosine residue is highly conserved among insect Met/Gce orthologs, and mutating it to phenylalanine reduced the amount of [^3^H]JH III bound to the Gce protein to 40%. This decrease likely reflects the reduced affinity of Gce either toward the epoxideless JH I derivative or MF relative to the epoxidated native JHs.

### Critical impact of the double-bond geometry on agonist activity

Ligand-binding, cell-based reporter, and *in vivo* gene activation and morphogenetic assays with JH I geometric isomers all yielded matching results that define the critical shape of an active Gce agonist. The data consistently ranked the activity of seven geometric isomers based on four rules. 1) The native 2*E*,6*E* conformation is the most active in all tests. 2) A single “twist” in the JH backbone markedly reduces all agonist activities. 3) Geometry is more critical at the 2,3 double bond than at the 6,7 double bond. 4) Altering the geometry at both double bonds renders an isomer inactive. It is gratifying to see that these rules apply, without exception, in the five diverse assays we performed with these compounds.

Our data agree remarkably well with early studies of the effect of JH geometry on inhibiting metamorphosis in diverse insect species. In the kissing bug, *Rhodnius prolixus*, the geometric isomers retained 7.3 (*E*,*Z*), 3.3 (*Z*,*E*), and 0.2% (*Z*,*Z*) of the potency of the native *E*,*E* conformation of 10*R*,11*S*-JH I (38), and virtually identical ratios were confirmed for *R. prolixus* and two other hemimetabolous species (39). This ranking very closely matches the different activities of these isomers recorded in our assays. Similar results were reported by Röller and Dahm (36) who also noted the critical role of the double-bond geometry at the 2,3-position. When tested for binding to an hJHBP from the moth *Manduca sexta*, the JH I isomers also ranked *E*,*E* > *E*,*Z* > *Z*,*E* > *Z*,*Z*, highlighting the importance of the 2,3 double bond for interaction with the carrier protein (43). The ratios between the association constants measured for hJHBP binding to the isomers again match our data.

The structure of Gce or Met has not yet been resolved, and our standard computational docking of ligands into a homology-based structural model of the PAS-B domain of Gce did not explain quantitatively why the unnatural geometric isomers, particularly *Z*,*Z*, cannot bind the receptor protein. It is intriguing that the same alterations in the geometry of the JH skeleton that affect the interaction of the hormone with the intracellular receptor Gce also affect JH I binding to the structurally dissimilar hemolymph JH-binding protein. This may suggest that particularly the *Z*,*Z* stereoisomer assumes a conformation that precludes its entry to the hormone-binding cavity or interaction with it, regardless of the nature of the ligand-binding pocket.

To address the problem more precisely, additional aspects have to be taken into account. For instance, rather than docking scores, one should attempt to determine Gibbs free energy of binding, which involves ligand internal flexibility and solvation/desolvation effects. While in the binding pocket, all ligands assumed similar conformations that did not correspond to their global energy minima. Based on the structural model of the binding and the calculated strain energies, we can conclude that the internal conformational barriers between the bound and unbound states of the ligands present a major factor discriminating between productive and nonproductive binding modes. If the difference between ligand conformations in bound and unbound states is large while most of the ligand–protein interactions are sustained, it is clear that Gibbs free energy of binding will primarily be determined by this difference. Only in one case of the moderately active stereoisomer *S*-(*E*,*Z*)-JH I was the strain energy similar to that calculated for the JH I and JH III native hormones, but *S*-(*E*,*Z*)-JH I apparently lost preferential noncovalent bonds to the protein cavity.

In summary, our present work reveals that the intracellular JH receptor Gce displays stereoselectivity toward its hormone agonists. Of greatest importance is the geometry of the JH skeleton where alteration is most critical at the 2,3 and better tolerated at the 6,7 double-bond position. Of moderate importance is the presence of the C10,C11 epoxide followed by the optical isomerism at the C10 and C11 positions. Contrasting with this exquisite stereoselectivity is the fact that Gce recognizes chemically disparate compounds such as the pyridine derivative pyriproxyfen (6) and the carbamate-based fenoxycarb or W330 (this study). Our data show that these compounds indeed act through the JH receptor Gce, and some exceed the activity of the native hormones. Full understanding of the structural basis of interaction of these potent JH mimics and insecticides with the JH receptor requires further research.

## Experimental procedures

### Reagents

Racemic *R*,*S*-[^3^H]JH III (10–20 Ci mmol^−1^) was purchased from PerkinElmer Life Sciences. *R*,*S*-JH III and fenoxycarb (ethyl *N*-[2-(4-phenoxyphenoxy)ethyl]carbamate) were from Sigma-Aldrich; methyl farnesoate (2*E*,6*E*) was obtained from Echelon Biosciences. The enantiomers of JH III (10*R* and 10*S*), separated using HPLC on a chiral stationary phase (51), were kindly donated by Dr. Tetsuro Shinoda. JH III bisepoxide was provided by Dr. José L. Maestro; we verified its identity and purity using NMR spectroscopy (supporting information). A series including the natural configuration of JH I with its stereoisomers (Fig. 1) and methyl (2*E*,6*E*,10*E*)-7-ethyl-3,11-dimethyltrideca-2,6,10-trienoate (a nonepoxidated version of JH I) were a generous gift from Dr. Karel Sláma; we verified their identity and purity using NMR (supporting information). Ethyl *N*-{2-{4-[(2-hydroxycyclohexyl)methyl]phenoxy}ethyl}carbamate (referred to as W330) was kindly provided by the author of the compound synthesis, Dr. Zdeněk Wimmer (55) (see supporting information for NMR analysis).

### Ligand-binding assays

The *D. melanogaster* Gce protein (amino acids 1–689; NCBI Reference Sequence NP_511160.1) with an N-terminal Myc epitope was expressed by *in vitro* transcription/translation from a codon-optimized DNA template in the *pK-Myc-C2* plasmid using the rabbit reticulocyte lysate TnT Quick T7 Coupled System (Promega) as described previously (6). The produced protein was divided into 15-μl aliquots to perform all measurements in triplicates. The protein aliquots were added to PEG-coated glass tubes containing ∼0.5 pmol (∼22,000 dpm) of *R*,*S*-[^3^H]JH III in 85 μl of binding buffer (20 mm Tris-HCl (pH 7.8), 5 mm magnesium acetate, 1 mm EDTA (pH 8), 1 mm DTT), and the 100-μl reaction was incubated at room temperature for 1 h. For competition assays, a constant amount of *R*,*S*-[^3^H]JH III was combined prior to protein addition with increasing input (0.0003–300 pmol) of a cold competitor.

For the competition assays, we modified a method previously used for the ecdysone receptor (61). Briefly, the entire volume of each reaction was applied to the center of a 25-mm Whatman GF/C glass fiber filter and allowed to adsorb for 30 s. The filter was then transferred onto a glass sinter vacuum manifold and washed immediately with 15 ml of the cold binding buffer (above) supplemented with 0.05% Nonidet P-40 detergent. Upon brief vacuum drying, filters were placed in scintillation vials with 7 ml of the UltimaGold XR (PerkinElmer Life Sciences) scintillation liquid, and the next day dpm was measured on a scintillation counter. To estimate input dpm, the entire volume of a reaction was spotted directly on a GF/C filter placed in a scintillation vial. The data were plotted as percentage of *R*,*S*-[^3^H]JH III bound against the molar concentration of a competing ligand, and *K_i_* values were calculated as described below (see “Data processing and statistics”).

For some *R*,*S*-[^3^H]JH III–binding assays and to complement data obtained with the above glass filter method, we used adsorption of unbound ligand to dextran-coated charcoal (DCC) (62), which we had previously adopted for JH receptor studies (5, 6). In this case, 20 μl of a DCC suspension (10 mm Tris-HCl (pH 7.5), 1.5 mm EDTA, 1% dextran, 5% Norit A) was added to the 100-μl binding reaction, incubated for 2 min, and centrifuged for 3 min at 12,000 × *g*. The supernatant (100 μl) was collected for scintillation counting. The glass filter and the DCC methods yielded fully consistent results.

### Luciferase reporter assay

*D. melanogaster* S2 cells were grown at 26 °C in Shields and Sang M3 insect medium (Sigma-Aldrich) supplemented with 0.5 g/liter KHCO_3_, 8% fetal bovine serum (Life Technologies), 100 units/ml penicillin, and 100 μg/ml streptomycin. The assay was based on a JH-inducible firefly luciferase reporter (*JHRE-luc*) driven by eight tandem repeats of a JHRE from the *A. aegypti early trypsin* gene (12) and was performed as described previously (6). Briefly, S2 cells seeded in 24-well plates were transfected with *JHRE-luc* (125 ng/well) and plasmids encoding the *Renilla* luciferase (50 ng/well) and the *D. melanogaster* Tai protein (125 ng/well) using FuGENE HD DNA transfection reagent (Promega). For RNAi experiments, double-stranded RNAs (1 μg/well) targeting either *gce*, *Met*, or *tai* mRNAs (*egfp* for control) were prepared as described previously (6) and included in the transfection mixture. Thirty-six hours post-transfection, cells were treated with a JH agonist dissolved in ethanol and incubated for an additional 8 h. Cells were then processed with the Dual-Luciferase System (Promega), and the luminescence readout from an Orion II microplate luminometer (Berthold) was recorded. Relative luciferase activity was normalized to *Renilla* luminescence, and EC_50_ values were determined as described below (see “Data processing and statistics”).

### Two-hybrid assay

An assay that measures ligand binding–dependent dimerization of JH receptor proteins with their partner Tai was recently developed by Miyakawa and Iguchi (48) based on the CheckMate Mammalian Two-Hybrid System (Promega). We obtained the described vectors expressing the *D. melanogaster* proteins Gce, Met (both in *pACT*), and a C-terminally truncated Tai (in *pBIND*) and reproduced the assay in the human HEK293T cell line. The HEK293T cells were grown at 37 °C under a 5% CO_2_ atmosphere in Dulbecco's modified Eagle's medium containing 10% fetal bovine serum (Life Technologies), 100 units/ml penicillin, and 100 μg/ml streptomycin. Cells seeded in 24-well plates were transfected using FuGENE HD (Promega) with a 200 ng/well *pG5luc* reporter and 50 ng/well *pACT-gce* (or *pACT-Met*) and *pBIND-Tai*, which also encodes *Renilla* luciferase. Thirty-six hours post-transfection, cells were treated with a JH agonist in ethanol and incubated for another 8 h before EC_50_ values were determined from the relative luciferase/*Renilla* activity as described below (see “Data processing and statistics”).

### Induction of Kr-h1 mRNA expression in vivo

Tested compounds were topically applied (100 μg in 0.25 μl of acetone per individual) using a Burkhard microapplicator on *D. melanogaster* at the white puparium stage; controls received 0.25 μl of acetone only. Twelve white puparia were used per treatment, and 24 h later they were pooled by three to obtain four replicates representing each treatment. The puparia were immediately frozen and subjected to total RNA extraction using TRI Reagent (Sigma-Aldrich) following the manufacturer's protocol. RNA was treated with TURBO RNase-free DNase (Ambion), and 2-μg RNA aliquots were used for first-strand cDNA synthesis using the SuperScript III kit (Invitrogen) with random hexamer primers. Transcripts were quantified using a LightCycler 480 qRT-PCR System (Roche Applied Science) with SYBR Green fluorescent label and previously described primers specific for *Kr-h1* and the ribosomal protein 49 (*rp49*) genes (6). All biological samples were examined in two technical replicates. To enable comparisons among all samples, a calibrator cDNA was applied to a master mix for specific genes on each plate, and *Kr-h1* expression was normalized relative to levels of *rp49*.

### Effects on Drosophila morphogenesis

For testing the agonist potential *in vivo*, we used the established white puparium bioassay (53). The tested compounds were delivered to white puparia in doses of 0.01, 0.1, 1.0, 10.0, and 100.0 μg per individual in 0.25 μg of acetone as described above. A total of 15–20 puparia represented each treatment. The animals were checked daily until all controls (treated with solvent only) had emerged as adult flies.

### Data processing and statistics

Results were plotted, and all the statistics were calculated using the Prism graphic program (version 6.0; GraphPad Software, San Diego, CA). In competition ligand-binding assays, *K_i_* values were determined using nonlinear regression analysis with the “one-site-fit *K_i_*” equation: log EC_50_ = log(10 log *K_i_* × (1 + concentration in nm of [^3^H]JH III/*K_d_* of [^3^H]JH III] binding to the Gce protein)); *Y* = Bottom + (Top − Bottom)/(1 + 10 (*X* − log EC_50_)). The *K_d_* of [^3^H]JH III binding to Gce was previously established as 19.27 nm (6). To determine EC_50_ values in the *JHRE-luc* and two-hybrid experiments, the data were processed using nonlinear regression (least squares ordinary fit) with the “sigmoidal dose-response (variable slope)” equation: *Y* = Bottom + (Top − Bottom)/(1 + 10 ((log EC_50_ − *X*) × Hill slope)). The values are given in the corresponding figure legends.

### Molecular modeling

The *D. melanogaster* Gce PAS-B protein sequence was retrieved from the UniProt database (UniProt accession number Q9VXW7). The model was created with the homology module of MOE software (63) (http://www.chemcomp.com)^5^ using the crystal structure of the hypoxia-inducible factor 2α PAS-B domain (Protein Data Bank^TM^ code 3F1P) (64) as the template for modeling; the template shares 48% sequence similarity with Gce PAS-B. A similar protocol was used in previous modeling of *T. castaneum* Met PAS-B (5). The protein was further processed using the Protein Preparation Wizard tool in Schrödinger (65–68). The hydrogen atoms were added, and partial charges were assigned. Hydrogen positions were refined using the restrained minimization (OPLS2005) method. Furthermore, ligand 2D schemes of JH molecules were generated using MOE and converted to 3D structures (63). The structures were prepared, and a conformational search to determine ligand minimum energy was performed using the LigPrep in Schrödinger (69). The lowest-energy conformer of the prepared ligands was utilized for the docking studies. The PAS-B–binding pocket was constructed by manual selection of composing amino acids reported from previous studies (5, 6). The grid was generated at the active site residues of the PAS-B domain, and docking was performed with the Glide program in Schrödinger (70). The binding energy was calculated using the MMGBSA application in Prime and determined as: MMGBSA Δ*G* bind(NS) = MMGBSA Δ*G* bind − Receptor strain − Ligand strain where MMGBSA Δ*G* bind = Complex − Receptor − Ligand. The important parameter is the Prime MMGBSA ligand strain energy. To obtain this value, the ligand was extracted from the optimized complex, and an energy calculation was run on it without minimization to find the energy of the ligand as optimized in the binding pocket. Next, energy minimization was run on the ligand outside of the receptor. Both calculations were done on the ligand alone in solution. The energy difference is the ligand strain energy.

## Author contributions

L. B., P. J., J. V., R. H., and M. J. conceptualization; L. B., R. H., and M. J. funding acquisition; L. B., P. J., M. D., P. K., J. V., R. H., and M. J. investigation; L. B., P. J., M. D., P. K., J. V., and M. J. methodology; L. B., R. H., and M. J. writing-original draft; P. J., M. D., and M. J. validation; J. V., R. H., and M. J. supervision; R. H. and M. J. resources; R. H. and M. J. writing-review and editing; M. J. project administration.

## Supplementary Material

Supporting Information

## Footnotes

5 Please note that the JBC is not responsible for the long-term archiving and maintenance of this site or any other third party-hosted site.

## Footnotes

4 The abbreviations used are:

20E: 20-hydroxyecdysone
JH: juvenile hormone
JHRE: JH response element
JHB3: JH III bisepoxide
MF: methyl farnesoate
Gce: germ cell–expressed
Met: methoprene-tolerant
Tai: Taiman
Kr-h1: Krüppel homolog 1
bHLH-PAS: basic helix-loop-helix/Per-Arnt-Sim
hJHBP: hemolymph JH-binding protein
MMGBSA: molecular mechanics, the generalized Born model and solvent accessibility
*JHRE-luc*: JH-responsive luciferase
EC_50_: effective concentration
*K_i_*: inhibition constant
mJHBP: mosquito juvenile hormone-binding protein
DCC: dextran-coated charcoal
S2: Schneider 2
rp49: ribosomal protein 49
qRT-PCR: quantitative RT-PCR.
