# Supplementary material for: Exquisite ligand stereoselectivity of a Drosophila juvenile hormone receptor contrasts with its broad agonist repertoire
Source: J Biol Chem. 2018 Nov 19;294(2):410–23. doi: 10.1074/jbc.RA118.005992 (PMC6333893; doi:10.1074/jbc.RA118.005992)
Supplement: Supporting Information [file supp_294_2_410__index.html]

Exquisite ligand stereoselectivity of a Drosophila juvenile hormone receptor contrasts with its broad agonist repertoire — JH receptor selectivity for activating ligands — Supporting Information 

# Exquisite ligand stereoselectivity of a *Drosophila* juvenile hormone receptor contrasts with its broad agonist repertoire

## Supporting Information

- Supporting Information (to be published online) - Table S1, NMR data, Figures S1, S2, S3, and S4, References
