# Supplementary material for: Exquisite ligand stereoselectivity of a Drosophila juvenile hormone receptor contrasts with its broad agonist repertoire
Source: J Biol Chem. 2018 Nov 19;294(2):410–23. doi: 10.1074/jbc.RA118.005992 (PMC6333893; doi:10.1074/jbc.RA118.005992)
Supplement: Supporting Information [file supp_RA118.005992_140961_1_supp_228903_phk90p.pdf]

SUPPLEMENTARY INFORMATION

Exquisite ligand stereoselectivity of a *Drosophila* juvenile hormone receptor contrasts with its broad agonist repertoire

Lenka Bittova[1,#], Pavel Jedlicka[2,#], Martin Dracinsky[2], Palani Kirubakaran[2], Jiri Vondrasek[2], Robert Hanus[2,§,*], and Marek Jindra[1,§,*]

From the [1]Biology Center of the Czech Academy of Sciences, Institute of Entomology, Ceske Budejovice 370 05, Czech Republic; [2]Institute of Organic Chemistry and Biochemistry of the Czech Academy of Sciences, Prague 166 10, Czech Republic

[#]These authors have contributed equally.
[§]Jointly corresponding authors.

*To whom correspondence should be addressed:
Marek Jindra, Biology Center, Czech Academy of Sciences, Branisovska 31, Ceske Budejovice 37005, Czech Republic; jindra@entu.cas.cz; Tel. +420-387775232
and
Robert Hanus, Institute of Organic Chemistry and Biochemistry, Czech Academy of Sciences, Flemingovo nám. 2, Prague 16610, Czech Republic; robert.hanus@uochb.cas.cz; Tel. +420-220183581

MATERIAL INCLUDED

NMR identification of the tested compounds
- **Figure S1**
- **Table S1**

Figure S2
Figure S3
Figure S4
References

NMR identification of the tested compounds

Identity and purity of the tested compounds was verified by NMR spectroscopy, using a Bruker Avance III 600 and/or Avance III 500 spectrometers (600.13 or 499.88 MHz for ^{1}H and 150.90 or 125.71 MHz for ^{13}C) equipped with a 5 mm PFG cryoprobe. All spectra were acquired for samples in $CDCl_3$ and referenced to TMS. Signals of all hydrogen and carbon atoms were assigned by using a combination of 1D and 2D (H,HCOSY, H,C-HSQC, and H,C-HMBC) techniques. Selected two- and three-bond H–C correlations observed in H,C-HMBC spectrum of native *S*-(*E*,*E*)-JH I are shown in Figure S1. These correlations allow for an unequivocal assignment of all ^{1}H and ^{13}C signals and confirmation of the compound's identity. ^{1}H and ^{13}C spectra of *S*-(*E*,*E*)-JH I are identical to those reported previously (1). Carbon chemical shifts of all carbon atoms in the series of JH I isomers are summarized in Table S1. A comparison of selected indicative chemical shifts in the series enabled unambiguous determination of the configuration of the double bonds and relative configuration on the chiral centre in position 11.

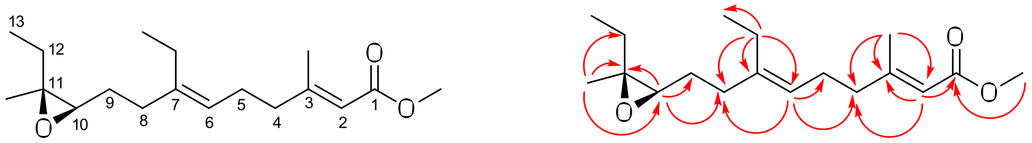

Figure S1. Atom numbering (left) and selected two- and three-bond H–C correlations in H,C-HMBC spectra (right) depicted on the structure of *S*-(*E*,*E*)-JH I.

Table S1. Chemical shifts of carbon atoms in JH I isomers. Indicative chemical shifts for configuration determination are colored. Atom numbering is shown in Figure S1.

Position	Compound (configuration)							Configuration	
	S-(*E*,*E*)-JH I (11*S*,2*E*,6*E*)	*S*-(*Z*,*E*)-JH I (11*S*,2*Z*,6*E*)	*S*-(*E*,*Z*)-JH I (11*S*,2*E*,6*Z*)	*S*-(*Z*,*Z*)-JH I (11*S*,2*Z*,6*Z*)	*R*-(*Z*,*E*)-JH I (11*R*,2*Z*,6*E*)	*R*-(*E*,*Z*)-JH I (11*R*,2*E*,6*Z*)	*R*-(*Z*,*Z*)-JH I (11*R*,2*Z*,6*Z*)		
**C1**	167.2	166.7	167.3	166.7	166.7	167.2	166.7		
**C2**	115.3	115.8	115.3	115.8	115.9	115.3	115.8		
**C3**	160.0	160.4	159.9	160.4	160.3	159.9	160.4		
**C4**	41.2	33.7	41.2	33.6	33.7	41.1	33.7	2*E*	2*Z*
**C5**	25.6	26.3	25.7	26.4	26.3	25.7	26.4		
**C6**	122.9	123.5	122.6	123.2	123.6	122.5	123.2		
**C7**	141.3	140.8	141.1	140.6	140.8	141.0	140.6		
**C8**	33.3	33.2	27.2	27.1	33.1	27.0	26.9	6*E*	6*Z*
**C9**	27.2	27.2	27.3	27.4	27.5	27.7	27.8		
**C10**	64.7	64.7	64.6	64.7	63.1	62.9	63.0	11*S*	11*R*
**C11**	61.8	61.8	61.9	61.9	61.7	61.7	61.7		
**C12**	25.8	25.8	25.8	25.7	31.5	31.4	31.4	11*S*	11*R*
**C13**	9.7	9.7	9.7	9.7	9.3	9.2	9.2	11*S*	11*R*

***S*-(*E*,*E*)-JH I**

methyl (2*E*,6*E*)-7-ethyl-9-[(2*R*,3S)-3-ethyl-3-methyloxiran-2-yl]-3-methylnona-2,6-dienoate

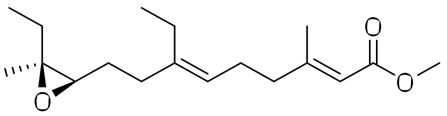

^{1}H NMR ($CDCl_3$) δ: 0.97 (t, 3H, $J_{CH3,CH2}$ = 7.6, 7-CH_2-$\underline{CH_3}$), 1.00 (t, 3H, $J_{13,12}$ = 7.6, H-13), 1.27 (s, 3H, 11-CH_3), 1.44–1.68 (m, 4H, H-9 and H-12), 2.01–2.06 (m, 2H, 7-$\underline{CH_2}$-CH_3), 2.07–2.22 (m, 6H, H-4, H-5 and H-8), 2.16 (d, 3H, $J_{CH3,2}$ = 1.3, 3-CH_3), 2.71 (dd, 1H, $J_{10,9}$ = 6.9 and 5.4, H-10), 3.68 (s, 3H, OCH_3), 5.09 (m, 1H, H-6), 5.67 (m, 1H, H-2).

***S*-(*Z*,*E*)-JH I**

methyl (2*Z*,6*E*)-7-ethyl-9-[(2*R*,3*S*)-3-ethyl-3-methyloxiran-2-yl]-3-methylnona-2,6-dienoate

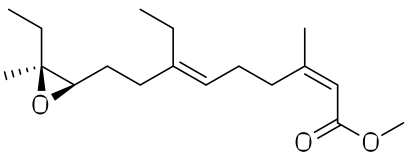

^{1}H NMR ($CDCl_3$) δ: 0.97 (t, 3H, $J_{CH3,CH2}$ = 7.6, 7-CH_2-$\underline{CH_3}$), 1.00 (t, 3H, $J_{13,12}$ = 7.6, H-13), 1.27 (s, 3H, 11-CH_3), 1.44–1.67 (m, 4H, H-9 and H-12), 1.89 (d, 3H, $J_{CH3,2}$ = 1.4, 3-CH_3), 2.01–2.21 (m, 6H, H-5, H-8 and 7-$\underline{CH_2}$-CH_3), 2.65 (m, 2H, H-4), 2.72 (dd, 1H, $J_{10,9}$ = 6.8 and 5.6, H-10), 3.67 (s, 3H, OCH_3), 5.16 (bt, 1H, $J_{6,5}$ = 7.1, H-6), 5.66 (m, 1H, H-2).

***S*-(*E*,*Z*)-JH I**

methyl (2*E*,6*Z*)-7-ethyl-9-[(2*R*,3*S*)-3-ethyl-3-methyloxiran-2-yl]-3-methylnona-2,6-dienoate

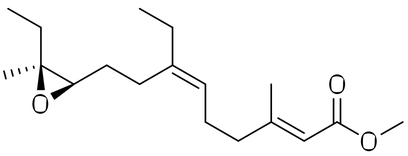

^{1}H NMR ($CDCl_3$) δ: 0.99 (t, 3H, $J_{CH3,CH2}$ = 7.4, 7-CH_2-$\underline{CH_3}$), 1.00 (t, 3H, $J_{13,12}$ = 7.6, H-13), 1.28 (s, 3H, 11-CH_3), 1.44–1.65 (m, 4H, H-9 and H-12), 1.99–2.04 (m, 2H, 7-$\underline{CH_2}$-CH_3), 2.12–2.23 (m, 6H, H-4, H-5 and H-8), 2.17 (d, 3H, $J_{CH3,2}$ = 1.3, 3-CH_3), 2.71 (dd, 1H, $J_{10,9}$ = 7.0 and 5.5, H-10), 3.69 (s, 3H, OCH_3), 5.11 (m, 1H, H-6), 5.67 (m, 1H, H-2).

***S*-(*Z*,*Z*)-JH I**

methyl (2*Z*,6*Z*)-7-ethyl-9-[(2*R*,3*S*)-3-ethyl-3-methyloxiran-2-yl]-3-methylnona-2,6-dienoate

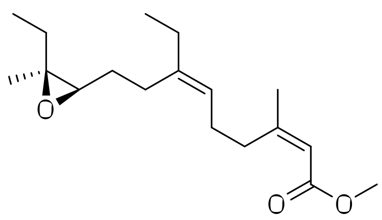

^{1}H NMR ($CDCl_3$) δ: 0.98 (t, 3H, $J_{CH3,CH2}$ = 7.5, 7-CH_2-$\underline{CH_3}$), 1.00 (t, 3H, $J_{13,12}$ = 7.6, H-13), 1.27 (s, 3H, 11-CH_3), 1.45–1.65 (m, 4H, H-9 and H-12), 1.89 (d, 3H, $J_{CH3,2}$ = 1.4, 3-CH_3), 1.98–2.03 (m, 2H, 7-$\underline{CH_2}$-CH_3), 2.13–2.25 (m, 6H, H-5 and H-8) 2.66 (m, 2H, H-4), 2.72 (dd, 1H, $J_{10,9}$ = 6.9 and 5.6, H-10), 3.67 (s, 3H, OCH_3), 5.19 (bt, 1H, $J_{6,5}$ = 7.2, H-6), 5.66 (m, 1H, H-2).

***R*-(*Z*,*E*)-JH I**

methyl (2*Z*,6*E*)-7-ethyl-9-[(2*R*,3*R*)-3-ethyl-3-methyloxiran-2-yl]-3-methylnona-2,6-dienoate

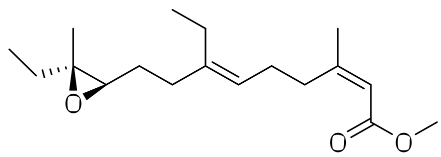

^{1}H NMR ($CDCl_3$) δ: 0.94 (t, 3H, $J_{13,12}$ = 7.5, H-13), 0.97 (t, 3H, $J_{CH3,CH2}$ = 7.6, 7-CH_2-$\underline{CH_3}$), 1.24 (s, 3H, 11-CH_3), 1.41–1.69 (m, 4H, H-9 and H-12), 1.89 (d, 3H, $J_{CH3,2}$ = 1.4, 3-CH_3), 2.02–2.21 (m, 6H, H-5, H-8 and 7-$\underline{CH_2}$-CH_3), 2.65 (m, 2H, H-4), 2.71 (t, 1H, $J_{10,9}$ = 6.3, H-10), 3.67 (s, 3H, OCH_3), 5.16 (bt, 1H, $J_{6,5}$ = 7.2, H-6), 5.66 (m, 1H, H-2).

***R*-(*E*,*Z*)-JH I**

methyl (2*E*,6*Z*)-7-ethyl-9-[(2*R*,3*R*)-3-ethyl-3-methyloxiran-2-yl]-3-methylnona-2,6-dienoate

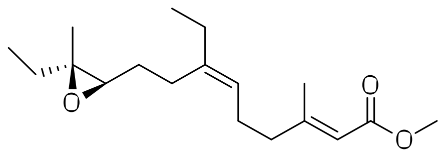

^{1}H NMR ($CDCl_3$) δ: 0.95 (t, 3H, $J_{13,12}$ = 7.5, H-13), 0.99 (t, 3H, $J_{CH3,CH2}$ = 7.5, 7-CH_2-$\underline{CH_3}$), 1.24 (s, 3H, 11-CH_3), 1.44–1.64 (m, 4H, H-9 and H-12), 1.99–2.03 (m, 2H, 7-$\underline{CH_2}$-CH_3), 2.10–2.23 (m, 6H, H-4, H-5 and H-8), 2.16 (d, 3H, $J_{CH3,2}$ = 1.3, 3-CH_3), 2.70 (dd, 1H, $J_{10,9}$ = 6.7 and 5.9, H-10), 3.68 (s, 3H, OCH_3), 5.11 (m, 1H, H-6), 5.67 (m, 1H, H-2).

***R*-(*Z*,*Z*)-JH I**

methyl (2*Z*,6*Z*)-7-ethyl-9-[(2*R*,3*R*)-3-ethyl-3-methyloxiran-2-yl]-3-methylnona-2,6-dienoate

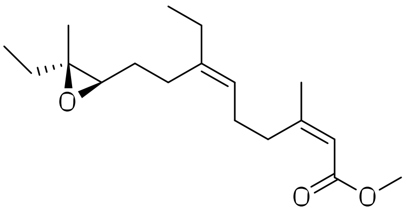

^{1}H NMR ($CDCl_3$) δ: 0.94 (t, 3H, $J_{13,12}$ = 7.5, H-13), 0.98 (t, 3H, $J_{CH3,CH2}$ = 7.4, 7-CH_2-$\underline{CH_3}$), 1.23 (s, 3H, 11-CH_3), 1.43–1.66 (m, 4H, H-9 and H-12), 1.89 (d, 3H, $J_{CH3,2}$ = 1.4, 3-CH_3), 1.98–2.03 (m, 2H, 7-$\underline{CH_2}$-CH_3), 2.11–2.23 (m, 4H, H-5 and H-8) 2.65 (m, 2H, H-4), 2.71 (t, 1H, $J_{10,9}$ = 6.3, H-10), 3.66 (s, 3H, OCH_3), 5.19 (bt, 1H, $J_{6,5}$ = 7.2, H-6), 5.66 (m, 1H, H-2).

deoxy-JH I

methyl (2E,6E,10E)-7-ethyl-3,11-dimethyltrideca-2,6,10-trienoate

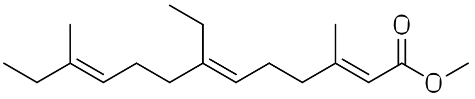

^{1}H NMR ($CDCl_3$) δ: 0.96 (t, 3H, $J_{CH3,CH2}$ = 7.6, 7-CH_2-$\underline{CH_3}$), 0.98 (t, 3H, $J_{13,12}$ = 7.5, H-13), 1.59 (s, 3H, 11-CH_3), 1.95–2.08 (m, 8H, H-8, H-9, H-12 and 7-$\underline{CH_2}$-CH_3), 2.17 (d, 3H, $J_{CH3,2}$ = 1.2, 3-CH_3), 2.15–2.20 (m, 4H, H-4 and H-5), 3.68 (s, 3H, OCH_3), 5.05 (m, 1H, H-6), 5.09 (m, 1H, H-10), 5.67 (m, 1H, H-2).

^{13}C NMR ($CDCl_3$) δ: 12.8 (C-13), 13.2 (7-CH_2-$\underline{CH_3}$), 15.9 (11-$\underline{CH_3}$), 18.9 (3-$\underline{CH_3}$), 23.2 (7-$\underline{CH_2}$), 25.6 (C-5), 26.7 (C-9), 32.3 (C-12), 36.5 (C-8), 41.3 (C-4), 50.8 (OCH_3), 115.2 (C-2), 122.4 (C-6), 122.7 (C-10), 136.9 (C-11), 142.1 (C-7), 160.2 (C-3), 167.3 (C-1).

JHB3

methyl (*E*)-5-(3-(2-(3,3-dimethyloxiran-2-yl)ethyl)-3-methyloxiran-2-yl)-3-methylpent-2-enoate

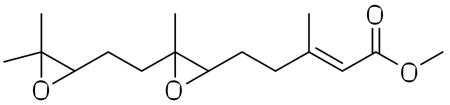

The sample is a mixture of diastereoisomers. ^{1}H NMR ($CDCl_3$) δ: 1.27–1.31 (m, 9H, 7-CH_3 and H-12), 1.52–1.80 (m, 6H, H-5, H-8 and H-9), 2.18 (d, 3H, $J_{CH3,2}$ = 1.3, 3-CH_3), 2.23–2.37 (m, 2H, H-4), 2.68–2.74 (m, 2H, H-6 and H-10), 3.69 and 3.69 (2 x s, 3H, OCH_3), 5.70–5.72 (m, 1H, H-2).

^{13}C NMR ($CDCl_3$) δ: 16.4 and 16.7 (7-$\underline{CH_3}$), 18.6, 18.7 and 18.8 (CH_3), 24.5 and 24.7 (C-9), 24.8 (CH_3), 26.7 (C-5), 35.2 and 35.9 (C-8), 37.6 (C-4), 50.9 (OCH_3), 60.5 and 60.6 (C7 and C11), 62.3 and 62.9 (C-10), 63.8 and 64.0 (C-6), 115.7 and 115.7 (C-2), 158.8 and 158.8 (C-3), 167.0 (C-1).

W330

ethyl N-{2-{4-[(2-hydroxycyclohexyl)methyl]phenoxy}ethyl}carbamate (2)

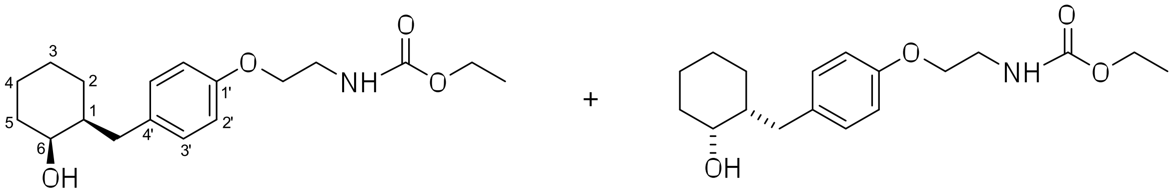

^{1}H NMR ($CDCl_3$) δ: 1.16–1.26 (m, 4H, CH_3 and H-5a), 1.34–1.47 (m, 4H, H-3a, H-4a and H-6), 1.53–1.71 (m, 3H, H-1, H-4b and H-5b), 1.76 (m, 1H, H-3b), 2.48 (dd, 1H, J_{gem} = 13.6, $J_{CH2,1}$ = 7.7, 4'-CH_2a), 2.65 (dd, 1H, J_{gem} = 13.6, $J_{CH2,1}$ = 7.5, 4'-CH_2b), 3.57 (q, 2H, $J_{CH2,CH2}$ = $J_{CH2,NH}$ = 5.3, NH-C$\underline{H_2}$), 3.78 (m, 1H, H-2), 4.01 (t, 2H, $J_{CH2,CH2}$ = 5.1, NH-CH_2-C$\underline{H_2}$), 4.12 (q, 2H, $J_{CH2,CH3}$ = 7.1, C$\underline{H_2}$-CH_3), 5.13 (bs, 1H, NH), 6.81 (m, 2H, H-2'), 7.10 (m, 2H, H-3').

^{13}C NMR ($CDCl_3$) δ: 14.6 (CH_3), 20.3 (C-4), 25.3 (C-5), 26.3 (C-6), 33.2 (C-3), 37.7 (4'-$\underline{C}H_2$), 40.5 (NH-$\underline{C}H_2$), 43.6 (C-1), 60.9 (CH_2-CH_3), 67.0 (NH-CH_2-CH_2), 68.4 (C-2), 114.2 (C-2'), 130.1 (C-3'), 133.5 (C-4'), 156.6 (C1').

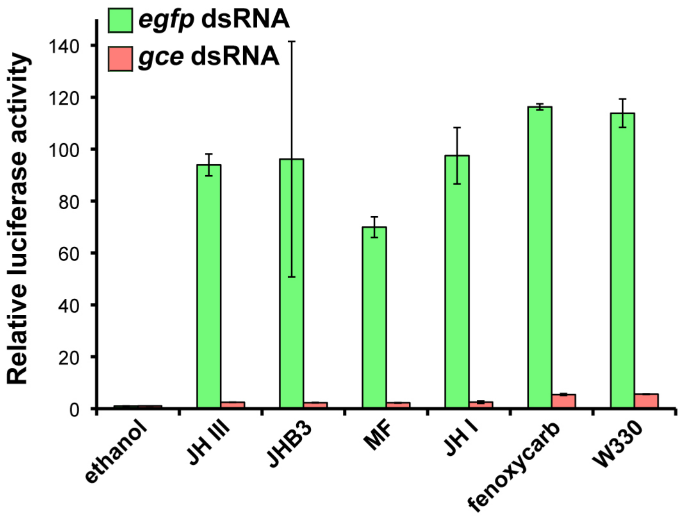

Figure S2. Activation of the *JHRE-luc* reporter in the *Drosophila* S2 cell line by both native insect juvenile hormones and their synthetic carbamate mimics requires the JH receptor Gce. S2 cells were co-transfected with the *JHRE-luc* reporter, a *Renilla* plasmid (for normalization), and 1 µg of either control (*egfp*) or *gce* double-stranded RNA per well in a 24-well plate. Cells were treated with the indicated agonists and processed with the Dual-Luciferase system (Promega) 8 h later. To ensure maximum response, MF was added at 10 µM concentration; all other agonists were applied at 1 µM concentration. Data are mean values from three experiments, plotted as fold increase of normalized luciferase activity relative to the value measured with solvent (ethanol) alone, which was arbitrarily set to 1. Error bars represent S.D. For more detail of Gce, Met and Tai requirement for the response to JH III and MF, see our previous report (3).

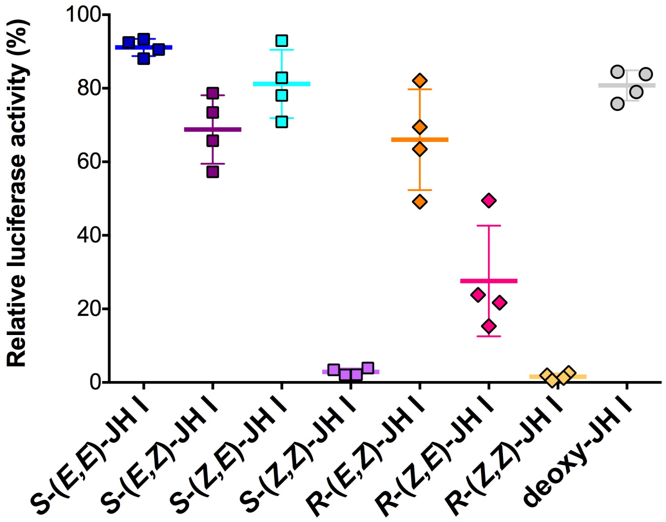

Figure S3. Activation of the *JHRE-luc* reporter in the *Drosophila* S2 cell line by the indicated compounds at 10 µM concentration. The data are mean values calculated from four independent experiments (each in three technical replicates); error bars represent S.D.

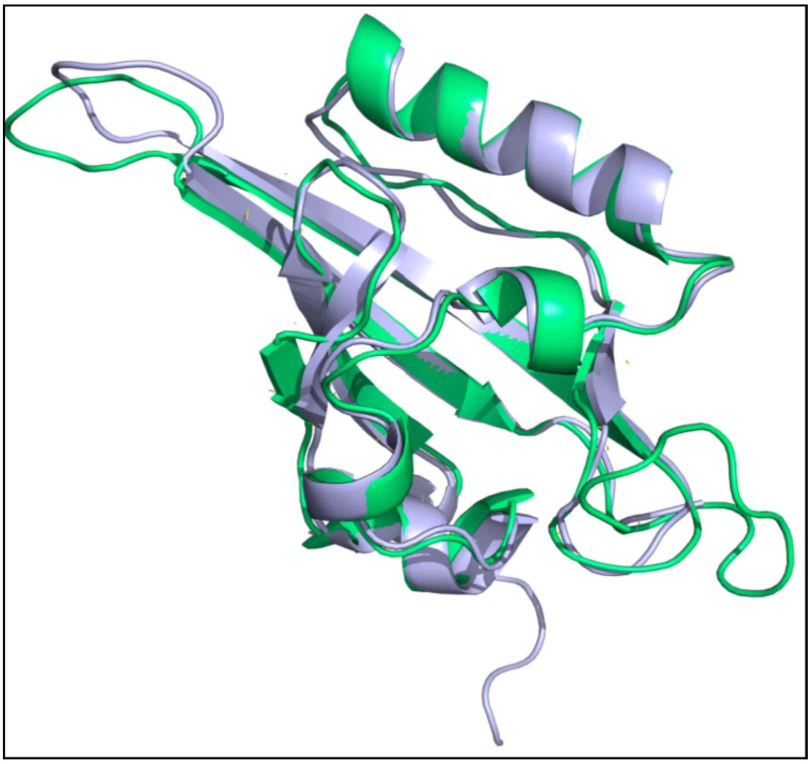

Figure S4. A homology structural model of the ligand-binding domain of the Gce protein. A model of the PAS-B domain was generated based on homology with the crystal structure (PDB ID: 3F1P) of the PAS-B domain of hypoxia-inducible factor 2α (HIF2α) (4). The region starting from Leu-262 (NCBI reference sequence NP_511160.1) of *Drosophila melanogaster* Gce (green) is superimposed with a previously developed model (5) of the JH receptor Met from the beetle *Tribolium castaneum* (light blue).

References

1. Okochi, T., and Mori, K. (2001) New enantioselective synthesis of (10*R*,11*S*)-(+)-juvenile hormones I and II. *Eur. J. Org. Chem.* **11**, 2145-2150
2. Wimmer, Z., Rejzek, M., Zarevucka, M., Kuldova, J., Hrdy, I., Nemec, V., and Romanuk, M. (1997) A series of bicyclic insect juvenile hormone analogs of Czech origin: Twenty years of development. *J. Chem. Ecol*. **23**, 605–628
3. Jindra, M., Uhlirova, M., Charles, J.-P., Smykal, V., and Hill, R. J. (2015) Genetic evidence for function of the bHLH-PAS protein Gce/Met as a juvenile hormone receptor. *PLoS Genet*. **11**, e1005394
4. Scheuermann, T. H., Tomchick, D. R., Machius, M., Guo, Y., Bruick, R. K., and Gardner, K. H. (2009) Artificial ligand binding within the HIF2α PAS-B domain of the HIF2 transcription factor. *Proc. Natl. Acad. Sci. U.S.A.* **106**, 450-455
5. Charles, J.-P., Iwema, T., Epa, V. C., Takaki, K., Rynes, J., and Jindra, M. (2011) Ligand-binding properties of a juvenile hormone receptor, Methoprene-tolerant. *Proc. Natl. Acad. Sci. U.S.A*. **108**, 21128–21133
